# A biomechanical comparison of conventional dynamic compression plates and string-of-pearls™ locking plates using cantilever bending in a canine Ilial fracture model

**DOI:** 10.1186/s12917-017-1139-8

**Published:** 2017-07-13

**Authors:** Allison R Kenzig, James R Butler, Lauren B Priddy, Kristen R Lacy, Steven H Elder

**Affiliations:** 1 Mississippi State University College of Veterinary Medicine, 240 Wise Center Drive, Mississippi State, MS 39762 USA; 2 Mississippi State University College of Agricultural and Biological Engineering, Box 9632, 130 Creelman Street, Mississippi State, MS 39762 USA

**Keywords:** SOP, String-of-pearls, DCP, Dynamic compression plate, Canine ilium, Ilial fracture, Plating, Biomechanics

## Abstract

**Background:**

Fracture of the ilium is common orthopedic injury that often requires surgical stabilization in canine patients. Of the various methods of surgical stabilization available, application of a lateral bone plate to the ilium is the most common method of fixation. Many plating options are available, each having its own advantages and disadvantages. The purpose of this study was to evaluate the biomechanical properties of a 3.5 mm String-of-Pearls™ plate and a 3.5 mm dynamic compression plate in a cadaveric canine ilial fracture model. Hemipelves were tested in cantilever bending to failure and construct stiffness, yield load, displacement at yield, ultimate load, and mode of failure were compared.

**Results:**

The mean stiffness of dynamic compression plate (116 ± 47 N/mm) and String-of-Pearls™ plate (107 ± 18 N/mm) constructs, mean yield load of dynamic compression plate (793 ± 333 N) and String-of-Pearls™ plate (860 ± 207 N) constructs, mean displacement at yield of dynamic compression plate (8.6 ± 3.0 mm) and String-of-Pearls™ plate (10.2 ± 2.8 mm) constructs, and ultimate load at failure of dynamic compression plate (936 ± 320 N) and String-of-Pearls™ plate (939 ± 191 N) constructs were not significantly different. No differences were found between constructs with respect to mode of failure.

**Conclusions:**

No significant biomechanical differences were found between String-of-Pearls™ plate and dynamic compression plate constructs in this simplified cadaveric canine ilial fracture model.

## Background

Fractures of the pelvis constitute approximately a quarter of all fractures in small animals [1–3]. The majority of these cases are due to high-energy trauma, such as vehicular trauma or falling from a height [1, 3–7]. Fractures of the ilial body account for 18–46% of pelvic fracture cases, with the majority being long oblique fractures, and they often occur concurrently with fractures of the ischium and pubis [1, 8]. Conservative management with cage rest may be utilized for minimally displaced fractures, but can result in sub-optimal long-term function if the fracture segments are displaced [3, 4]. Surgically treated ilial fractures are generally stabilized by open reduction and lateral application of appropriately contoured plates and screws [1, 4–8]. Various types of plates have been employed for the stabilization of ilial fractures, including dynamic compression plates, cuttable plates, T plates, miniplates, reconstruction plates, and double plates [4, 8].

The most common complication associated with ilial fracture repair is implant failure, which occurs in up to 62% of patients. The majority of these failures are due to screw pull-out, with one of the reasons for this high incidence of screw loosening being poor bone quality in the cranial ilial wing [1, 8–12]. Conventional plating techniques rely on friction between the plate and bone to provide stability. The weakest point in this conventional plating system is the interface between the screw and the bone. Placing screws in poor quality bone, such as the cranial ilium, may result in an inability to generate adequate force to prevent implant and fracture motion [1]. Furthermore, the amount of compression needed to generate friction between a standard compression plate and bone has been shown to adversely affect the periosteal blood supply, which has been linked to delayed healing, non-union, and increased susceptibility to bacterial surgical site infections after fracture repair [13, 14]. Locking implant systems have been developed to provide more stable fracture repair, especially in poorer quality bone, and to minimize the negative impact on local vascularity during fracture healing [1]. These plating systems do not rely on plate-to-bone friction to provide stability, eliminating the need for high shear loads at the screw-to-bone interface [15]. Less plate-to-bone contact also decreases the need for precise plate contouring and helps preserve the blood supply to the bone [13–17].

The String-of-Pearls™ (SOP) implant is a stainless steel locking plate made up of repeating units of spherical pearls and cylindrical rods. Each pearl is designed to engage a standard cortical bone screw in a locking fashion by the screw threads engaging a ridge within the pearl, and the plate can be contoured by twisting along its longitudinal axis and bending in multiple planes without compromising its locking ability. The ability of the SOP locking plate to utilize standard cortical bone screws allows this construct to, in general, be considerably less expensive than more traditional locking plate constructs [13, 14]. In a 2014 study of Locking Compression Plates (LCP) versus Dynamic Compression Plates (DCP) in a canine ilial fracture model, there was no demonstrable difference between the constructs’ performance in acute failure testing in vitro [1]. In a 2015 study comparing double SOP plating versus single DCP constructs in a synthetic bone model, the double SOP constructs had significantly greater bending stiffness, bending strength, bending structural stiffness, and torsional stiffness [13]. To the authors’ knowledge, there have been no studies reported in the literature comparing the mechanical strength of SOP plating to DCP plating in a canine ilial fracture model.

The purpose of this study was to compare the stiffness, yield load, ultimate load at failure, displacement at yield, and mode of failure in cantilever bending of SOP and DCP constructs in an acute failure ilial fracture model. The hypothesis was that the SOP plates would have superior biomechanical properties compared to the DCP constructs of comparable size.

## Methods

Pelves were harvested from 11 healthy, purpose-bred dogs,^1^ weighing approximately 20–30 kg, that were humanely euthanized for reasons unrelated to orthopedic disease or this study. The dogs were part of an institutional approved teaching protocol unrelated to this study. The pelves were harvested by disarticulation at the sacroiliac joints within 12 h of euthanasia and stripped of all associated soft tissues. Visual inspection of the samples was performed to ensure they were free of pre-existing orthopedic disease. The samples were then stored at −20 °C after being wrapped in saline soaked towels and double bagged.

**Fig. 1:**
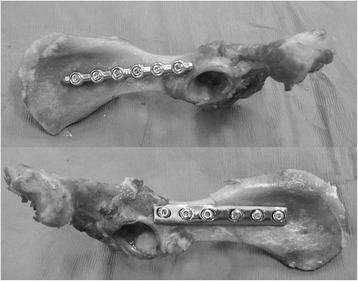
Lateral String-of-Pearls™ plate and Dynamic Compression Plate application demonstrating osteotomy location, plate contouring, and fracture reduction

**Fig. 2:**
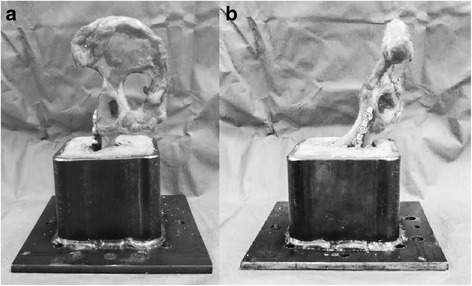
**a** Mounting of hemipelvis with pubic symphysis perpendicular to platform. **b** Mounting of the hemipelvis with the long axis oriented 30 degrees from perpendicular to the platform

**Fig. 3:**
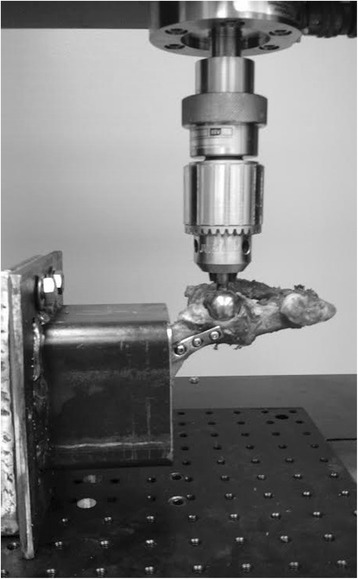
Mounting of hemipelvis on testing platform

Prior to biomechanical testing, the samples were thawed at room temperature. The pelves were divided at the symphysis, and the paired ilia were randomly assigned to treatment with either a stainless steel six-hole 3.5 mm DCP^2^ or a six-hole 3.5 mm SOP^3^ construct such that the treatment groups were evenly distributed with respect to side. For both plating techniques, the plate was positioned with the cranial aspect of the plate centered within the wing of the ilium and the caudal aspect of the plate adequately cranial to the acetabulum so that no screws penetrated the joint. Three self-tapping cortical screws^4^ were positioned cranial and three caudal to the osteotomy. For both plating systems, the plates were individually contoured to match the curvature of the respective ilium. Standardized long oblique osteotomies were performed using a sagittal saw. The osteotomy location was standardized at 40% of the distance from the cranial aspect of the acetabulum to the most cranial aspect of the ilial wing. Osteotomies were created such that the fracture line extended from the most caudal aspect of the ventral iliac spine to a point on the greater ischiatic notch which produced an osteotomy that was 1.5 times the length of the dorsoventral ilial shaft diameter. The osteotomies were then reduced, and the assigned constructs were applied using standard AO^5^ technique (Fig. 1). All holes were drilled with a variable speed, hand-held, battery-powered drill^6^ loaded with a 2.5 mm drill bit,^7^ and using either a DCP drill guide^8^ or an SOP drill guide^9^ of the appropriate size, for the corresponding plates. Drill holes were not tapped due to the use of self-tapping cortical screws^4^. The screws were tightened by hand until they were “two-finger-tight” for the DCP construct, and until the screws locked into the SOP plates. The two screws adjacent to the osteotomy were placed eccentrically in the DCP constructs in order to provide fracture compression. The cranial aspect of each ilium was mounted in a custom-made steel rectangular pot using polymethylmethacrylate^10^ such that pubic symphysis was perpendicular to the platform, and the long axis of the hemipelvis was 30 degrees from perpendicular to the platform (Fig. 2a and b). The plates and protruding ends of the screws were covered by a modelling compound^11^ so that the acrylic would not adhere to or contact the implants during testing. The modelling compound was removed prior to biomechanical testing. The potted bone was fitted to a servohydraulic materials testing machine.^12^ A 22 mm diameter stainless steel sphere^13^ was placed in contact with the acetabulum prior to testing and used to transmit the load from the acetabulum to the load cell (Fig. 3). The angle of the potted ilia allowed for testing the constructs at 120°, which approximates hip angle during midstance [18]. Constructs were mounted for a bending stress state and tested to failure using a compression loading frame with a 50 kN load cell.^14^ The constructs were preloaded to 5 N, and load was applied at 20 mm/min until failure was detected by an acute drop in load, concurrent with grossly detectable evidence of construct failure. Data were sampled at a rate of 100 Hz and stored electronically. Mode of failure, including screw pull-out, screw cut-out, and plate bending was recorded by observation during testing and inspection of the constructs after testing. Failure was classified as screw pull-out or screw cut-out when construct failure occurred with no observed bending of the plate. Screw cut-out occurred with damage to the surrounding bone, whereas screw pull-out occurred with no surrounding bony damage.

### Data analyses

For each sample, a load-displacement curve was generated to determine ultimate load, stiffness, displacement at yield, and yield load. The maximum force applied during testing was the ultimate load (N). A linear trendline was obtained for the elastic portion of the load-displacement curve, and the slope of the line was defined as stiffness (N/mm). A 1 mm displacement offset method was used to determine displacement at yield (mm) and yield load (N).

### Statistical analyses

Data were analyzed by paired t-tests and reported as mean ± standard error of the mean (SEM). Differences were considered significant when *p* < 0.05. All statistical testing was performed using a standard statistical software package.^15^

## Results

**Fig. 4:**
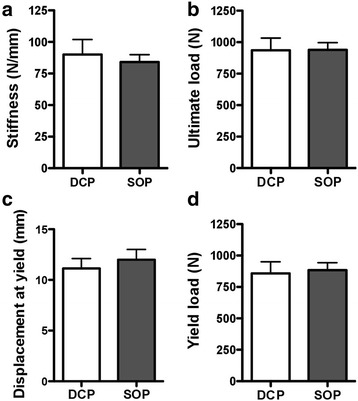
Comparison of stiffness (**a**), ultimate load (**b**), displacement at yield (**c**), and yield load (**d**) for DCP and SOP constructs

Five left hemipelves and 6 right hemipelves were plated with a DCP construct. Six left hemipelves and 5 right hemipelves were plated with an SOP construct. In the DCP group, constructs failed through either screw pull-out in the cranial ilium (*n* = 9) or through plate bending at the osteotomy site (*n* = 2). In the SOP group, the constructs failed through either screw cut-out cranial to the ilium (*n* = 7) or through plate bending at the osteotomy site (*n* = 4). Screw cut-out for the SOP group occurred with apparent fracture of the ilium surrounding the screws, as opposed to the DCP group for which screw pull-out occurred without damage to the surrounding bone. In both groups, plate bending occurred at the osteotomy site without obvious screw loosening. No significant differences were found between groups for stiffness, ultimate load at failure, displacement at yield, or yield load (Table 1 and Fig. 4).

**Table 1:** Comparison of stiffness, ultimate load, displacement at yield, and yield load for DCP and SOP constructs

	Stiffness (N/mm)	Ultimate load (N)	Displacement at yield (mm)	Yield load (N)
DCP	116 ± 47	936 ± 320	8.6 ± 3.0	793 ± 333
SOP	107 ± 18	939 ± 191	10.2 ± 2.8	860 ± 207
*p*-value	0.69	0.98	0.53	0.79

*DCP* dynamic compression plate, *SOP* String-of-Pearls™ plate. Values are reported as mean ± SEM

## Discussion

This study did not reveal any significant differences between SOP and DCP constructs in a cadaveric canine ilial fracture model in acute cantilever bending to failure. Previous studies comparing SOP plates, various forms of DCP constructs, and locking plate constructs have produced results that differ from the presented study. In 2008, DeTora and Kraus compared SOP, LCP, standard limited contact DCP (LC-DCP), and broad LC-DCP constructs. The results of that study showed that the broad LC-DCP was stronger in stiffness and bending compared to the SOP; however, the SOP was biomechanically superior to both the LCP and the LC-DCP [19]. In 2011, Blake et al. compared SOP to DCP, stainless steel and titanium LC-DCP, LCP, advanced locking plate system (ALPS), and Fixin plates in a validated bone model simulating bridging osteosynthesis. This study found that SOP constructs had significantly higher stiffness than all the other plates, and were significantly stronger than the titanium LC-DCP, ALPS, and Fixin constructs. The clinically relevant conclusion of this study stated that due to differing plate construct properties inherent to diverse implant systems, identical approaches to fracture management and plate application cannot be applied [20]. In 2012, Malenfant and Sod compared SOP to LCP constructs in a cadaveric tibial diaphyseal fracture gap model and found that the SOP construct was superior under bending static and cycling testing, but the LCP construct was superior in static and cycling torsion testing [21]. Hutcheson et al. compared bridging double plate SOP constructs to broad DCP constructs in a synthetic bone fracture gap model in 2015 and found that all biomechanical properties were significantly higher for the double SOP construct, but the actual differences were small [13].

The mechanical advantages of the SOP implant from the previous studies were often attributed to the circular profile of the implant and higher associated area moment of inertia compared to rectangular implants [13–15, 19–21]. The difference in fracture model configuration in the presented study compared to the multiple aforementioned studies likely allowed less demonstration of the previously observed biomechanical advantages of the SOP plating system. In our study the most common mode of failure was screw pullout from the cranial ilial wing. The diminished quality of bone in that portion of the ilium likely contributed to the lack of differences between the two constructs. Additionally, in previous studies, load application was purposefully oriented to examine the weakest plate direction. The current ilial fracture model tested the DCP oriented more with the width of the bone, rather than its thickness, resulting in an increase in the area moment of inertia, which places it at a mechanical advantage compared to the previous studies. The SOP plates are not affected by this change in force directionality as they are cylindrical implants.

A similar cadaveric ilial fracture model comparing 6-hole 3.5 mm DCP constructs to 3.5 mm LCP constructs also found no significant difference between the two constructs [1]. The anatomically reduced fracture permitted load sharing within the constructs and was one explanation of the minimal difference seen [1]. The interfragmentary compression achieved in DCP constructs likely benefited the DCP group in our study and may be an additional explanation for the lack of differences seen between constructs. Should interfragmentary compression not be clinically possible, the DCP construct *may* be weaker. However, additional mechanical studies would be required to validate that claim. The inherent characteristics of the SOP locking plate, including ease of contouring and decreased impact on vascularity due to decreased plate-to-bone contact, remain potential benefits despite the biomechanical similarity to the DCP constructs.

The oblique ilial osteotomy used in this fracture model is a similar method to the 2008 study by Fitzpatrick et al. which compared lateral ilial fracture plating to pelvic external skeletal fixators [4]. This fracture configuration was chosen due to its clinically relevant nature. Fractures of the ilium are most commonly long oblique fractures of the midbody with the obliquity oriented from cranioventral to caudodorsal [3, 5–8]. While it is known that the tension surface of the ilium is the ventral surface, and ventral plate fixation offers mechanical advantages compared to a lateral plate, we elected to evaluate lateral plating as the lateral surface of the ilium is more easily approached and is the most common method of internal fixation of pelvic fractures in dogs [1, 21–23].

Limitations of this study included the use of a cadaveric model and testing that was limited to cantilever bending. While cyclic testing would potentially provide more clinically applicable data, it is difficult to accurately reproduce the in vivo environment in a cadaveric model. To approximate the forces exerted on clinically repaired ilia, we elected to use a cantilever-bending model and tested the constructs at an angle that approximated midstance. However, the fracture model tested does not account for the various fracture configurations or patient conformations that may be encountered clinically. Fracture callus formation and bone remodeling following implant application may differ in SOP compared to DCP constructs due to the aforementioned effects on vascularity, which may in turn affect overall construct stability and/or healing rates. However, interfragmentary compression achieved with DCP constructs may also accelerate healing. This fracture model also does not account for potentially improved screw purchase with engaging the sacral wing, which has been studied previously with conflicting results [9, 10].

## Conclusions

In this study, no significant differences were found in stiffness, yield load, ultimate load, displacement at yield, or mode of failure between 3.5 mm SOP and DCP ilial fracture constructs. Further studies, including further in vitro studies evaluating cyclical testing and the effect of sacral screw purpose, and prospective clinical trials, would be necessary to further evaluate any true mechanical or clinical benefit to SOP fixation compared to DCP fixation for oblique mid-body ilial fractures in the dog.

## Abbreviations

ALPS: Advanced locking plate system
DCP: Dynamic compression plate
LC-DCP: Limited contact dynamic compression plate
LCP: Locking compression plate
SEM: Standard error of the mean
SOP: String-of-Pearls™

## Footnotes

1 Oak Hill Genetics, Ewing, Illinois, USA

2 Dynamic Compression Plate (DCP): Orthomed, Halifax, West Yorkshire, UK

3 String-of-Pearls™ (SOP): Orthomed, Halifax, West Yorkshire, UK

4 3.5 mm cortical screws: Orthomed, Halifax, West Yorkshire, UK

5 Arbeitgemeinschaft für Osteosynthesefragen / AO Foundation

6 Makita drill: Makita, La Mirada, California, USA

7 2.5 mm drill bit: Veterinary Orthopedic Implants, Saint Augustine, Florida, USA

8 DCP drill guide: DePuy Synthes, West Chester, Pennsylvania, USA

9 SOP drill guide: Orthomed, Halifax, West Yorkshire, UK

10 Technovit: Jorgensen Labs, Loveland, CO, USA

11 Play-Doh: Hasbro, Pawtucket, RI, USA

12 MTI-2 K Universal Testing Machine: Measurements Technology, Inc., Marietta, GA

13 316 Stainless Steel Precision Ball 7/8″ Diameter, product number 96415 K87: McMaster-Carr, Douglasville, GA, USA

14 Universal Testing Instrument, Model 1011: Instron, Norwood, MA, USA

15 GraphPad Prism 5: GraphPad Software, Inc., La Jolla, CA, USA
